# An efficient approach to detect and track winter flush growth of litchi tree based on UAV remote sensing and semantic segmentation

**DOI:** 10.3389/fpls.2023.1307492

**Published:** 2023-11-30

**Authors:** Shudai Bai, Juntao Liang, Teng Long, Changjiang Liang, Jinxin Zhou, Weiyi Ge, Binshan Huang, Yubin Lan, Jing Zhao, Yongbing Long

**Affiliations:** ^1^ College of Electronic Engineering/College of Artificial Intelligence, South China Agricultural University, Guangzhou, China; ^2^ National Center for International Collaboration Research on Precision Agricultural Aviation Pesticides Spraying Technology, Guangzhou, China; ^3^ Country Guangdong Laboratory for Lingnan Modern Agriculture, Guangzhou, China

**Keywords:** semantic segmentation, UAV, litchi, flush tracking, quantitative analysis

## Abstract

The immature winter flush affects the flower bud differentiation, flowering and fruit of litchi, and then seriously reduces the yield of litchi. However, at present, the area estimation and growth process monitoring of winter flush still rely on manual judgment and operation, so it is impossible to accurately and effectively control flush. An efficient approach is proposed in this paper to detect the litchi flush from the unmanned aerial vehicle (UAV) remoting images of litchi crown and track winter flush growth of litchi tree. The proposed model is constructed based on U-Net network, of which the encoder is replaced by MobeilNetV3 backbone network to reduce model parameters and computation. Moreover, Convolutional Block Attention Module (CBAM) is integrated and convolutional layer is added to enhance feature extraction ability, and transfer learning is adopted to solve the problem of small data volume. As a result, the Mean Pixel Accuracy (MPA) and Mean Intersection over Union (MIoU) on the flush dataset are increased from 90.95% and 83.3% to 93.4% and 85%, respectively. Moreover, the size of the proposed model is reduced by 15% from the original model. In addition, the segmentation model is applied to the tracking of winter flushes on the canopy of litchi trees and investigating the two growth processes of litchi flushes (late-autumn shoots growing into flushes and flushes growing into mature leaves). It is revealed that the growth processes of flushes in a particular branch region can be quantitatively analysed based on the UAV images and the proposed semantic segmentation model. The results also demonstrate that a sudden drop in temperature can promote the rapid transformation of late-autumn shoots into flushes. The method proposed in this paper provide a new technique for accurate management of litchi flush and a possibility for the area estimation and growth process monitoring of winter flush, which can assist in the control operation and yield prediction of litchi orchards.

## 1 Introduction

Litchi, a traditional fruit crop in South China, has always been one of the most popular fruits because of its delicious flavour, attractive colour, and high nutritive value (Zhao et al., 2020). As the world’s rich litchi country, China ranks the first in litchi production (69.9% of total production) followed by Thailand, India, and Vietnam (Pareek, 2016; Qi et al., 2016). Due to the economic value of litchi fruit, there is interest in the physiology of this tropical species so that management techniques can be designed to maximize fruit yield.

**Figure 1:**
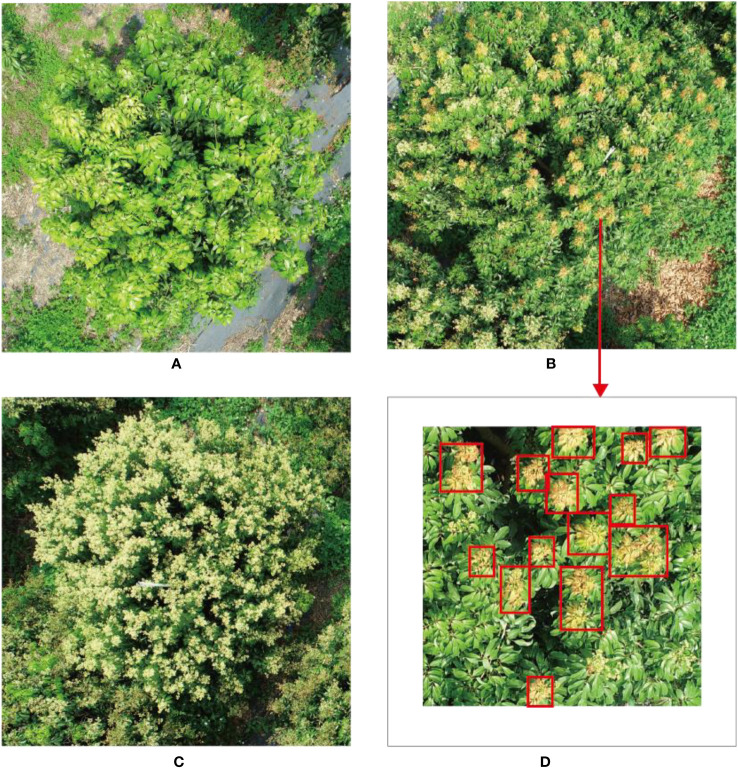
Litchi tree with different flowering rate. **(A)** Litchi tree with no flowers (the flushes have not grown into mature leaves before the flower bud differentiation stage). **(B)** Litchi tree with canopy partially covered by flowers (there are no flower spikes in the region where there are flushes). **(C)** Litchi tree with canopy fully covered by flowers (the flushes have grown into mature leaves before flower bud differentiation). **(D)** The red boxes denote flushes in panel **(B)**.

However, litchi’s unit yields are generally low and unstable (Xu et al., 2010), which is affected by various factors, such as soil tillage, fertilizer, climate and so on. Many studies have shown that in addition to the above external factors, the maturity of terminal shoots before flowering is the internal factor, which also directly affects the flowering rate, fruit setting rate and final litchi yield (Liu et al., 2022). In general, shoot growth has a rapid period of shoot elongation and leaf expansion. Leaf expansion needs to go through three different development stages of red, yellowish red and yellowish green leaves, and then enter the maturity period of dark green leaves (Hieke et al., 2002). Due to the suitable climate, litchi leaves can grow from red leaves to dark green leaves in autumn in September to October every year. The late-autumn shoots or winter shoots drawn in November to December, however, cannot grow into ripe green leaves on schedule because of the influence of temperature, and the colour of the leaves is still red, yellowish red and yellowish green. The occurrence of immature winter flush for litchi trees at flowering stage could result in relatively low flowering rate since the immature flush prevents the flower bud differentiation and then reduces the flowering rate on the canopy (see **Figure 1**). In litchi planting, leaves of the terminal shoots (potential flowering branches) should be dark green and matured during floral induction and differentiation stage, and winter flushes should be removed or killed (Yang et al., 2015) to ensure adequate energy for new shoots of leaves, floral buds, axillary buds at the beginning of spring (Chen et al., 2004) and then enhance the flowering rate. Therefore, in the management of litchi orchards, accurate monitor of flush growth in winter and efficient estimate of the area ratio of flush cannot only provide guidance for irrigation control in orchard management, but also produce more nutrient for the growth of flower buds. It plays an important role in the increase and prediction of the yield of litchi, and helps to enhance the market value of litchi.

Despite the importance of obtaining area proportion on flushes, there are still relatively few studies on automatic detection and segmentation of litchi flushes, and the progress towards this goal is still relatively slow. At present, estimating and controlling flushes still relies more on the manual judgment and operation of experienced orchard managers. However, the following questions inevitably arise: (1) Due to the large planting area of litchi orchards, the work of estimating and judging the area and growth of winter flushes is labour-intensive and time-consuming; (2) Because of the tall physiological characteristics of litchi trees, the winter flushes on the top of the trees are not observed by manual methods, which makes it difficult to monitor the growth of winter flush, and it is easy to obtain qualitative statistics on the area of winter flushes rather than quantitative statistics; (3) The proportion of winter flushes area of each litchi tree could not be counted by the artificial estimation method.

In recent years, UAV, as a technology to acquire high spatial and temporal resolution remote sensing images, has been widely used in precision agriculture because of its obvious advantages such as simple structure, strong mobility, high spatial and temporal resolution, synchronous acquisition of images and spatial information (Osco et al., 2021),which greatly reduces human resources and contributes to the improvement of crop production and efficiency (Aslan et al., 2022).A UAV with flexible movements and a camera provides basic support to manpower in situation assessment and surveillance application (Aslan et al., 2022), and advance in UAV technology provides producers with options for assessment of crucial factor impacting crop yield and quality, and also offers researchers with a non-destructive, objective manner for obtaining phenotypic measurements (Olson and Anderson, 2021). Torres-Sanchez et al. (2014) evaluated the accuracy, spatial and temporal consistency of six different vegetation indices (CIVE, ExG, ExGR, Woebbecke index, NGRDI and VEG) in a wheat crop using super resolution images from a low-cost camera attached to a UAV, and studied the effects of flight altitude (30 and 60 m) and days after seeding (DAS from 35 to 75DAS) on the accuracy of FVC classification. This study demonstrated that the low-cost-camera UAV can be used in precision agriculture applications, such as vegetation mapping in weed management.

Deep learning, which is rapidly gaining momentum as an image processing and data analysis method (Heaton, 2017), is an artificial neural network approach with multiple hidden layers and deeper combinations (Osco et al., 2021). As computer processing and labelling samples (i.e., samples) became more available, the performance of deep neural networks (DNNs) in image processing applications had improved, which tended to improve its performance and returned a larger learning capability than most common networks or other types of learners (Lecun et al., 2015; Ball et al., 2017). Although known for the high demand for computational power and high requirement for labelled data, deep neural networks have been successfully applied in agricultural applications such as weed detection, agricultural output assessment, etc.

The UAV remote sensing methods based on deep learning can be categorized into classification, object detection and segmentation tasks. Convolutional neural network and recurrent neural network are the most commonly used network architectures (Liu et al., 2021). In the study of agricultural precision management tasks, image segmentation technology is often used to obtain specific spatial characteristics and time-varying information of crop characteristics, because it provides basic information for crop growth monitoring, plant breeding evaluation, differentiation analysis and decision making (Hunt and Daughtry, 2018). For rice lodging recognition, Su et al. (2022) proposed a semantic segmentation method for rice lodging recognition that can process multi-band images by using UAV remote sensing images and combining U-Net network with dense block, dense network, attention mechanism and jump connection, which provided a useful reference for rice breeding and agricultural insurance claim settlement. The improved DeepLabv3 network is proposed for precise image segmentation of individual leaves by Yang et al. (2023), which demonstrates that the proposed method can thus effectively support the development of smart agroforestry. Genze et al. (2022) used three different state-of-the-art deep learning architectures combined with residual neural networks as feature extractor to develop a weed segmentation model for detecting weeds in sorghum fields under challenging conditions. The results showed that the U-Net architecture with ResNet34 feature extractor obtains more than 89% F1 scores on the persistence test set. Mo et al. (2021) proposed a convenient semi-automatic image annotation method and a high resolution digital ortho image segmentation method based on partition to segment the canopy images of fruit trees collected by UAV, which is of great significance for the accurate management of orchards. Anand et al. (2021) used a multiscale attention semantic segmentation method to automatically detect farmland anomalies. Kamal et al. (2022) used the fully convolutional network (FCN) and ResNet to analyse the accuracy of the weed and crop segment. A global accuracy of more than 90% in the verification package was achieved for both structures, which verified that FCN network can assist in agricultural weed and crop segmentation. Behera et al. (2023) deployed a lightweight convolutional neural network (CNN) architecture in real-life settings on a UAV that can be used in mapping urban areas, agricultural lands, etc.

Although UAV remote sensing and deep learning technologies are widely used in precision agriculture, there is no literature that applies these technologies to segmenting and tracking winter flushes. This paper aims to develop a new approach based on UAV technology combined with deep learning to segment the winter flush of litchi trees and then track the growth process of the flushes.

The main contributions of this paper can be summarized as follows: 1) A new litchi flush dataset was constructed for the training and verification of the litchi flush segmentation model. 2) An improved lightweight semantic segmentation model was proposed. With litchi flush as the research object, image semantic segmentation technology was used to segment litchi flush image, which achieved the expected target and segmentation effectiveness to solve the problem of extracting growth information of litchi flush in practical application. 3) The improved segmentation model was applied in the actual production of litchi orchard to estimate the area and track the growth process of winter flushes of litchi tree. The development stage and growth range of flushes were analysed in combination with temperature, and the changes in the area proportion of flushes in regional branches were quantitatively analysed, providing prediction information of early flush extraction and monitoring information on winter flush growth changes for accurate management of flush.

## 2 Materials and methods

### 2.1 Study areas

**Figure 2:**
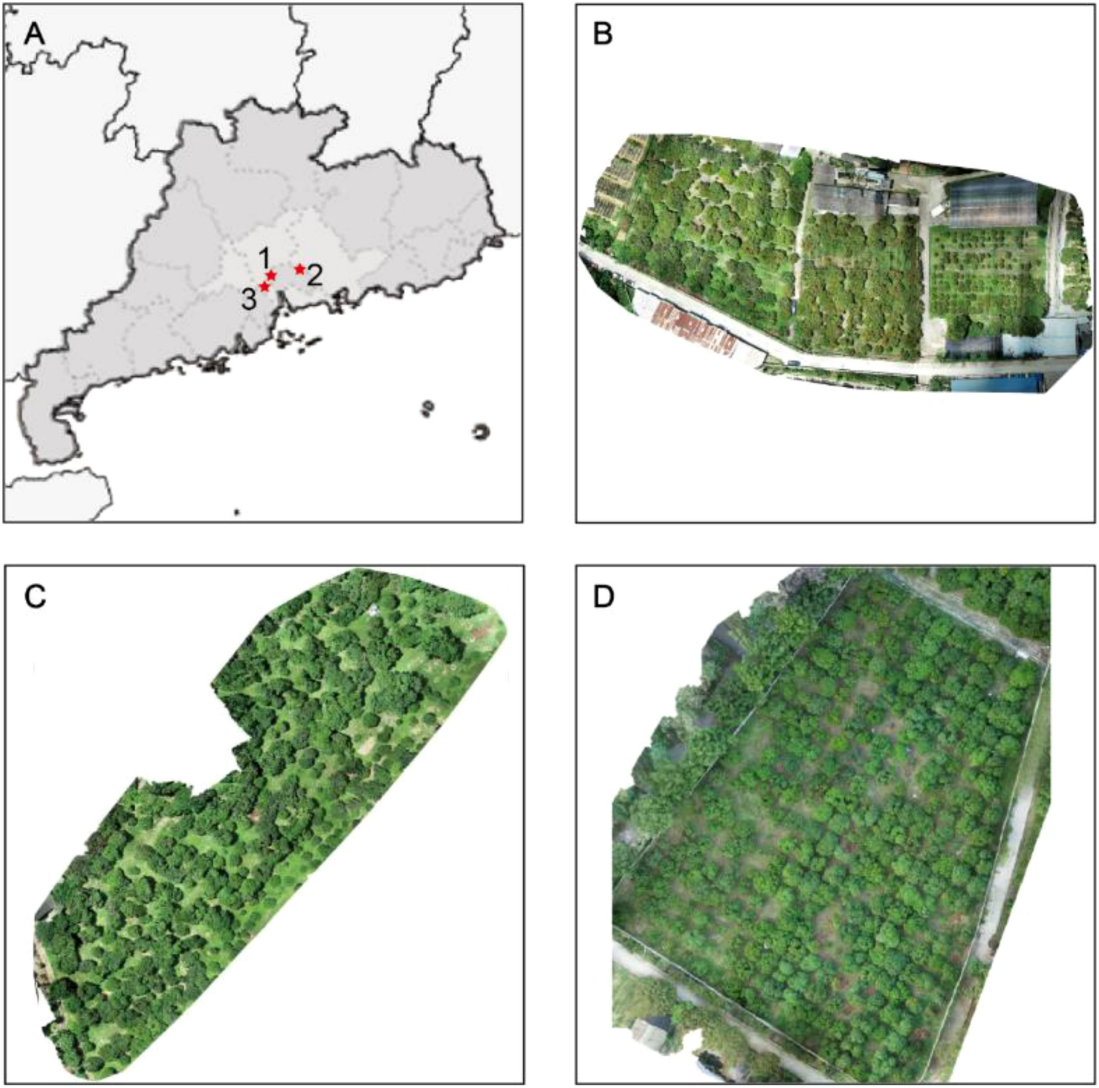
Overview of the study areas. **(A)** The locations of the study areas. **(B–D)** digital orthophoto maps of Areas A, B, and C acquired by a UAV, respectively.

The UAV remote sensing image of flushes for litchi trees are collected in three litchi orchards in Guangzhou, as is shown in **Figure 2**. The orchards are denoted as Area 1, 2 and 3. Area 1 is the Xianjinfeng planting area of Fruit Tree Research Institute of Guangdong Academy of Agricultural Sciences, located in Tianhe District (113°22′44.68″E, 23°9′29.05″N). Area 2, No. 9 of Mache New Fruit Farm, locates in Zengcheng District (113°47′7.43″E, 23°14′37.65″N). The main varieties panting in Area 2 are Guiwei and Nuomici. Area 3 is the litchi Garden of South China Agricultural University, located in Tianhe District (113°21′59.20″E, 23°14′37.65″N). There are 200 litchi trees with varieties such as Heiye and Feizixiao and so on.

### 2.2 UAV image collection

DJI Phantom 4 Pro is used to get images of three regions. The parameters for obtaining UAV remoting sensing images are as follows: Flight altitude: 8-20 meters, aerial image overlap: 30%, lens and ground Angle: 90°, image resolution: 5472 × 3648 pixels, flight speed: 2.5m/s, time: April to December 2022, from10:00am to 5:00pm. The specific experimental data collection information is shown in **Table 1**.

**Table 1:** The Aerial data details.

Experimental area	Number of images	Flight height(m)	Date of shooting	Variety
Fruit Research Institute, Academy of Agricultural Sciences	77	8	2022.04.03-04.18	Xianjinfeng,Guiwei
	211	10		
	136	15		
Mache New Fruit Farm	35	15	2022.05.17-10.21	Guiwei,Nuomici
	189	20		
South China Agricultural University Litchi Garden	94	10	2022.10.14-12.16	Heiye,Feizixiao
	65	15		

### 2.3 Data processing and labeling

In the data preprocessing, the unclear UAV remote sensing images and the images without litchi trees are removed from the dataset. When the original images with 5472×3648 pixels are fed into the deep learning network, it will cause memory overflow. Therefore, the original images in the litchi flush dataset are cropped into small images with a resolution of 512 × 512 pixels, and the small images without flushes are removed. Finally, 358 litchi flush images are obtained.

**Figure 3:**
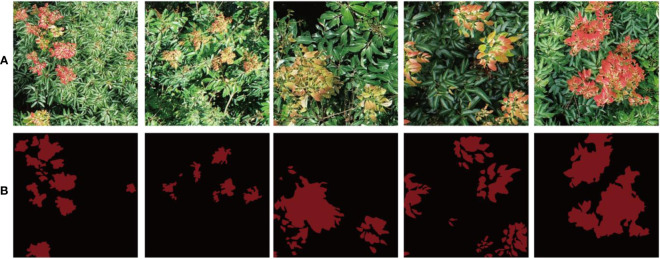
Image presentation of part of the flush dataset. **(A)** is the original image of 512 x 512 pixels. **(B)** is the label image of **(A)** object.

Since the supervised deep learning model is adopted for image segmentation in this paper, positive and negative samples need to be marked before image segmentation, so the region of interest (ROI) in the image needs to be manually marked. In this study, labelme is used to label the samples in the image, and the litchi flush samples are labeled as two items, flush and background. The label box is an irregular polygon around the ROI. The image annotation information for the flush and the background are generated into the corresponding Json file after the markup process. The Json file contains the image storage information, the image name, the comment name, and the coordinate information for the multiple deformations of each tag. **Figure 3** shows a partial image of dataset.

### 2.4 Semantic segmentation of litchi flush dataset

#### 2.4.1 U-Net

U-Net, a biomedical image semantic segmentation model proposed by Ronneberger et al. (2015), is designed to train end-to-end with a small number of images and generates more accurate segmentations. This makes it very suitable for agriculture yield because there are not enough labeled data to train complex CNN architectures (Lin et al., 2019). The model first performs well in biomedical image segmentation and subsequently outperforms earlier segmentation methods in many other areas (Ciresan et al., 2012). U-Net consists of two parts: encoder and decoder. The encoder is a typical convolutional network structure. Each module of the encoder contains two convolution layers and a pooling layer, and each convolution is followed by a ReLu activation function. This part down-samples the input image, captures its context, and outputs the coarse feature map. The decoder which has up-sampling layers receives the coarse feature map (produced by the encoder) and produces the final fine prediction image using transposed convolutions. This up-sampling process makes the output of the network the same size as the input image, thus achieving pixel-level segmentation. Copy and Crop section mediates between the encoder and decoder sections. It uses skip connections to connect the middle outputs of the encoder to the inputs of the middle layers of the decoder at the appropriate locations, that is, to fuse high-resolution information (texture information) and low-resolution information (position information). This connection process can fill in the underlying information to improve the segmentation accuracy.

#### 2.4.2 MobileNetV3

**Figure 4:**
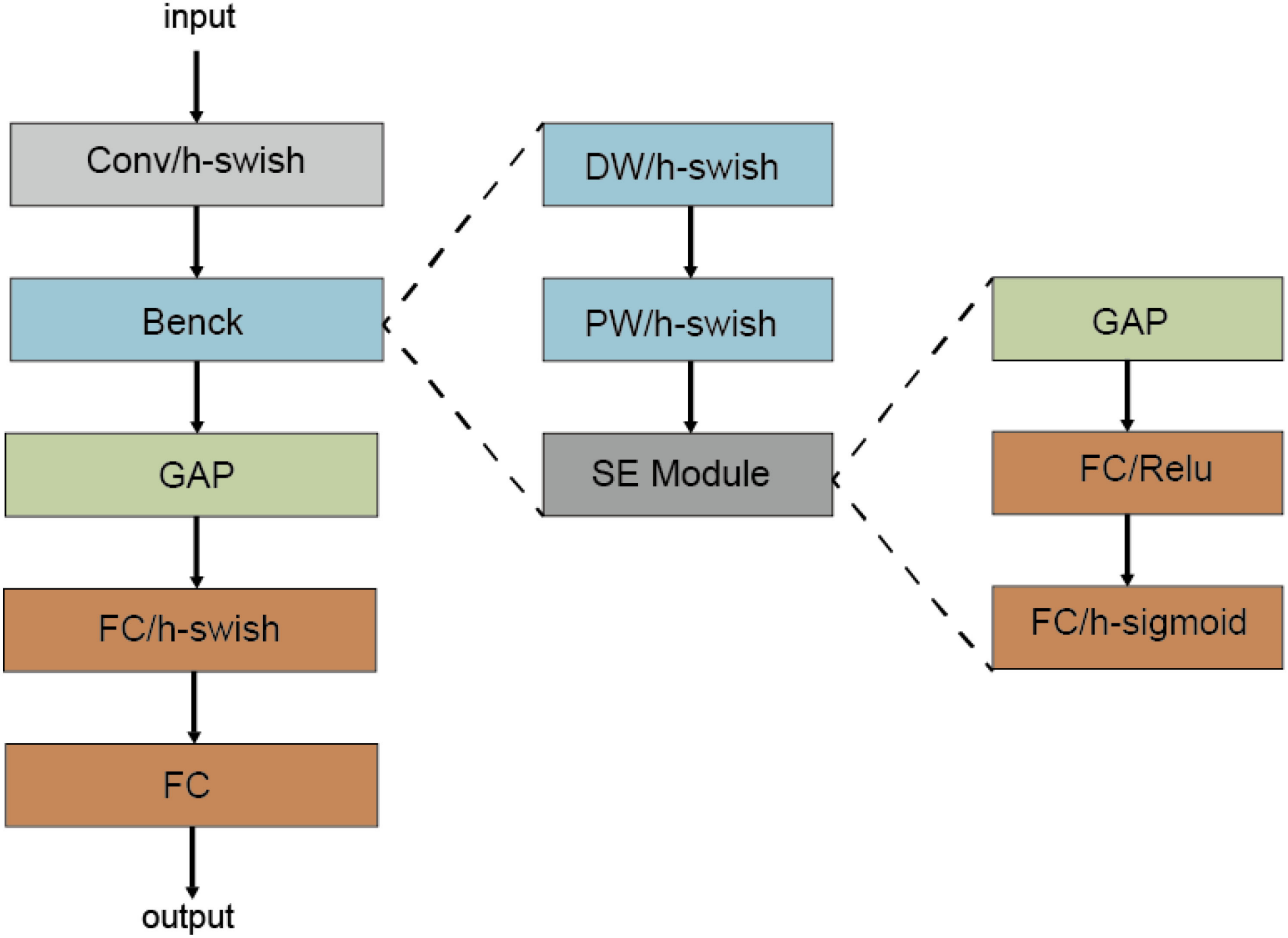
The network structure of MobileNetV3 model.

MobileNetV1 (Howard et al., 2017) introduces deep separable convolution as an effective alternative to traditional convolution layers. In the network structure, the first layer is the standard convolution layer, and the others are deep separable convolution. A 7×7 average pooling layer is connected after convolution, and then through a fully connected layer. Finally, the output of the fully connected layer is normalized by using the Softmax activation function to get the result. MobileNetV2 (Sandler et al., 2019) introduces Linear Bottlenecks and Inverted Residuals. The bneck structure is a reverse residual structure consisting of a 1x1 extended convolution, a deep convolution and a 1x1 projection layer, with the last convolution using a linearly activated bottleneck structure instead of the ReLu function. The structure of MobileNetV3 (Howard et al., 2019) is shown in **Figure 4**. Based on MobileNetV2, SE module is first added into the bneck structure and h-swish nonlinear activation function is used, and then Neural Architecture Search parameter (NAS) is used. Finally, the number of cores in the first convolution layer is reduced, the Last Stage is streamlined, and the time-consuming layer structure is redesigned. MobileNetV3 is defined as two models: MobilenetV3-Large and MobilenetV3-small. These models target high resource and low resource usage, respectively.

#### 2.4.3 Transfer learning

Deep learning often requires large-scale data to train and optimize the network model, but in the research on litchi flush segmentation, the number of images with pixel-by-pixel annotation tends to be small. Overfitting problems often occur when network models are trained on a small dataset. At the same time, the marking of litchi flush data image is time-consuming and tedious, and it is subject to subjective influence. Transfer learning can solve the problem of poor training of deep convolutional neural network model caused by small amount of data. MobileNetV3 network is a lightweight convolutional neural network focused on mobile terminals or embedded devices. Compared with traditional convolutional neural networks, it greatly reduces model parameters and computation on the premise of a small decrease in accuracy, and obtains sufficient training on large image datasets to learn a large number of features required for image classification and recognition. Therefore, transfer learning idea (Zhuang et al., 2021) can be applied to optimize litchi flush segmentation model by making full use of the large amount of knowledge learned from MobileNetV3 pre-training model. A common transfer learning method is feature transfer, which removes the last layer of the pre-training network and sends its previous activation value (which can be regarded as feature vector) to classifier such as support vector machine for classification training; The other is parameter migration, which only needs to re-initialize a few layers of the network (such as the last layer), and the remaining layers directly use the weight parameters of the pre-trained network, and then use the new dataset to fine-tune the network parameters. This paper adopted the transfer learning mode of feature transfer, and used MobileNetV3 as the feature extractor of image segmentation. By replacing the backbone part of U-Net, MobilenetV3_unet was finally modified to be used for litchi flush segmentation. Compared with the new learning (that is, randomly initialize the weight parameters of all layers of the network and use the training data set to start the new training of the network from the beginning), the introduction of the pre-trained network model is helpful to accelerate the training speed and improve the accuracy.

#### 2.4.4 Attentional mechanism module

When the decoder is used to extract litchi flushes information, the extraction ability of high-dimensional features is weakened due to the need of up-sampling. At the same time, cascade structure of U-Net does not distinguish the usefulness of the information (Su et al., 2022). In this paper, an attention mechanism was added to the cascade structure of the U-Net decoder (Hu et al., 2018) as well as the up-sampling process, and the use of available information was enhanced by adaptive control of the weight of each channel. This attention mechanism includes both channel attention and spatial attention. Given a high-resolution remote sensing image, it will produce a multichannel feature map F∈RC×H×W (where C, H and W denote the number of channels, the height, the width of the feature map, respectively) after passing through several convolutional layers. The information expressed in the feature map of each channel is different. Channel attention aims to use the relationships between each channel of the feature map to learn a 1D weight W_c∈RC×1×1 , and then multiply it to the corresponding channel. Learning in the channel dimension in this manner can obtain the importance of each channel and pay more attention to the semantic information that is meaningful to the current task. Spatial attention converts the spatial information of the original image to another space and retains the key information through the spatial module, and generates a weight mask for each location and weights the output, thereby enhancing the area of interest for a specific target and weakening the irrelevant background. Therefore, the use of spatial attention helps to summarize spatial information, especially the spatial information of small objects. In this paper, the Squeeze-and-Excitation (SE) and Efficient Channel Attention (ECA) modules of channel attention mechanism and the Convolutional Block Attention Module (CBAM) module of channel & space attention mechanism are used respectively.

- SE: SE module does attention or gating operations on the channel dimension. This attention mechanism allows models to focus on the most informative channel features and suppress the unimportant channel features.
- ECA: ECA module is proposed by QL Wang et al. The local cross-channel interaction strategy without dimensionality reduction effectively avoids the impact of dimensionality reduction on channel attention learning.
- CBAM: The representative model of the mixed Attention mechanism is the CBAM, which uses the combination of channel attention module CAM and spatial attention module SAM to process the input feature layer respectively.

#### 2.4.5 Improved U-Net model: Mobileunet-CC

**Figure 5:**
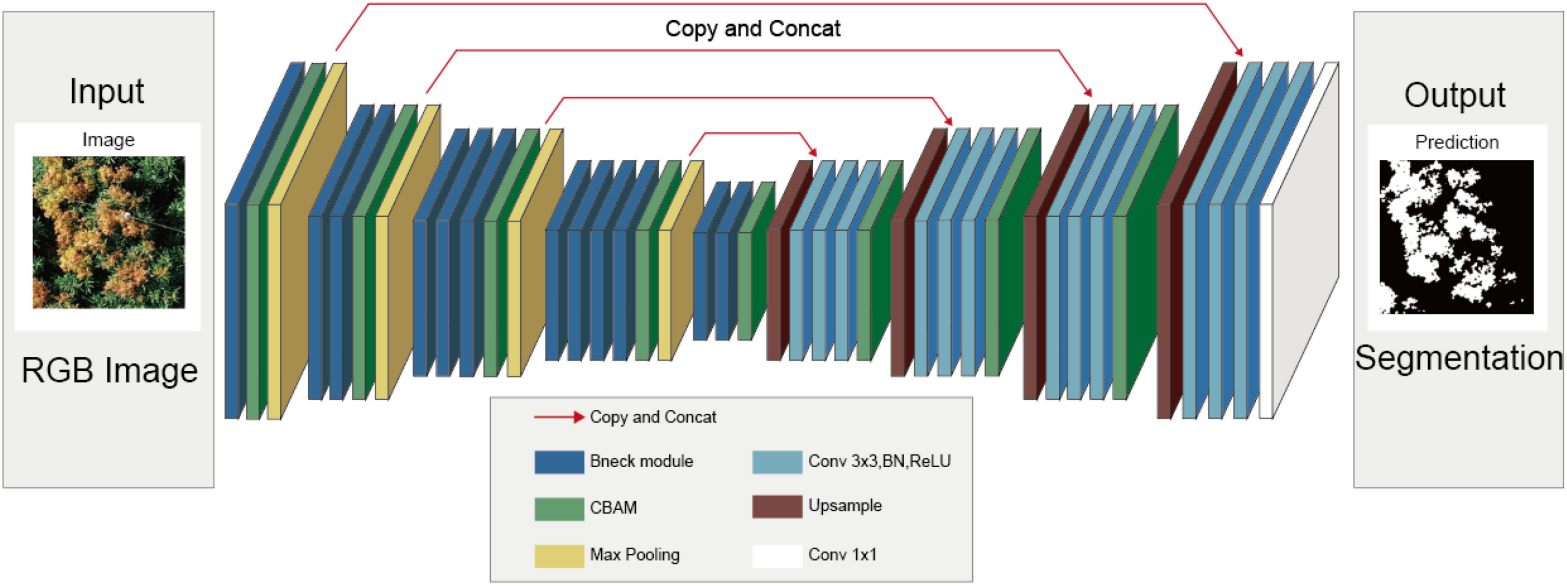
The network structure of Mobileunet-CC.

The network structure of the Mobileunet-CC model proposed in this paper is shown in the **Figure 5**. The model is constructed based on U-Net network, of which the encoder is replaced by MobeilNetV3 backbone network, one convolutional layer is added in each Double Conv module of the decoder and CBAM modules are integrated in both encoders and decoders. The bneck module contains multiple size and depth separable convolution blocks, batch normalization (BN) layers, and H-Switch activation function, etc. In addition, bneck modules with different depths and different quantities are used to extract higher-level image features according to the position of U-Net encoder part. The decoder upsamples the features through bilinear interpolation to compact the feature channels. The compressed features are fused with the same number of channels in the encoder, then the litchi flush features are extracted with three convolution kernel sizes of 3×3. CBAM is used to extract hierarchical features of images and enhance feature extraction capabilities of the model. The final feature segmentation map is output by a 1x1 convolution. The 3×3 convolutional layer in the original U-Net network does not use the padding 0 strategy, which makes the output size of the convolution smaller each time. Therefore, the padding strategy is used for each convolutional layer in the proposed network, where the 7×7 convolutional fill is 3 and the 3×3 convolutional fill is 1, which makes the size of the feature maps before and after the convolution consistent. In the section of experiment, the detailed segmentation results of the improved model are presented.

#### 2.4.6 Performance evaluation

Semantic segmentation is still a pixel classification problem, and the most commonly used loss function is the Cross Entropy Loss Function (CEL). But for litchi flush dataset, there is a problem of imbalance between positive and negative samples, which cannot be solved well by using cross entropy loss function. In this paper, Dice Loss function is used to replace the traditional cross entropy loss function, i.e.


(1) $$\begin{array}{c} L_{Dice}=1-\sum_{k=1}^{K}\frac{2w_{i}\sum_{i=1}^{N}p\left(k,i\right)g\left(k,i\right)}{\sum_{i=1}^{N}p\left(k,i\right)+\sum_{i=1}^{N}g\left(k,i\right)} \end{array}$$

In addition, pixel Accuracy (PA), Intersection over Union (IoU) and dice coefficient are the performance metrics selected for validation of the proposed semantic segmentation model.

PA is the ratio of the correct pixels to the total pixels. The calculation formula is as follows:


(2) $$\begin{array}{c} PA=\sum_{i=1}^{k}\frac{P_{ii}}{\sum_{i=1}^{k}\sum_{j=1}^{k}P_{ij}} \end{array}$$

MIoU is the most commonly used evaluation index in semantic segmentation experimental research. First, calculate the ratio of the real and predicted values of the two sets of real and predicted values to the union, and then calculate the average value of all categories. The calculation formula is as follows:


(3) $$\begin{array}{c} MIoU=\frac{1}{k+1}\sum_{i=1}^{k}\frac{P_{ii}}{\sum_{j=1}^{k}P_{ij}+\sum_{j=1}^{k}P_{ji}-P_{ii}} \end{array}$$

## 3 Experiment and discussion

### 3.1 Flush segmentation experiment

This section discusses the training and result analysis of the semantic segmentation model of litchi flush. The 308 images in flush dataset are randomly divided into training set and verification set with a ratio of 8:2, that is, 246 images were used for training, and 62 images were used for confirmation. And another 50 images were used as the test set. The model training GPU is RTX 3060 and the CPU is AMD Ryzen9 5900HS with Radeon Graphics 3.30 GHz. Deep learning frameworks all used pytorch for semantic segmentation model training.

#### 3.1.1 Basic model selection

**Figure 6:**
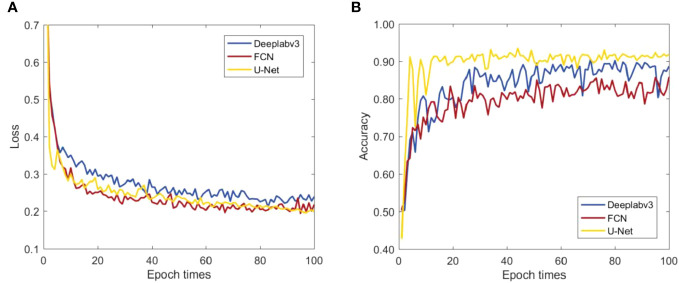
**(A)** Loss function and **(B)** pixel accuracy curves of the verification set.

**Figure 6** shows the loss function and the pixel accuracy curves for three network models: FCN (Long et al., 2015), Deeplabv3 (Chen et al., 2018), Deeplabv3 and U-Net. In **Figure 6A**, the loss function curve rapidly decreased in the initial epochs for all three models. In the subsequent process, the loss function of FCN model (yellow line) and Deeplabv3 model (blue line) had obvious fluctuations. For the U-Net model, however, the loss function curve decreased rapidly and steadily. The reason is that the litchi flush dataset is relatively small due to the production difficulty and it is not suitable for network structures with too many depth parameters such as Deeplabv3 and FCN, etc. As can be seen from **Figure 6B**, the pixel accuracy of U-Net model reaches the highest value of 90.95%, which is 0.3% and 5.15% higher than that of Deeplabv3 and FCN models, respectively. This happens because the skip connection and up-sampling structures of U-Net can obtain more feature information in the training set. In addition, the encoder and decoder structure of U-Net can help to better learn the characteristics of litchi flush dataset. Moreover, the shape, texture and other features of flushes in litchi flush dataset are relatively fixed and similar, and the data amount is small. The characteristic of the flush dataset is similar to the medical image, where U-Net performs excellent performance. As a result, better performance can be expected for the U-Net performs in flush dataset. Based on the above comparison, it can be concluded that U-Net is more suitable to be selected as the basic network model for constructing flush segmentation models.

#### 3.1.2 Ablation experiment

Since there are variable modules in Mobileunet-CC model, the comparison experiment is carried out to ensure that all the module in the model is optimal. It mainly includes the following three experiments: 1) Comparing the results of different feature extraction structures; 2) Comparing the effects of different attention mechanisms on performance; 3) Comparing the effect of pre-training weights.

MobilenetV3 network (Howard et al., 2017) uses deep detachable convolution and introduces the SE module and h-swish. VGG16 network (Simonyan and Zisserman, 2014) has smaller filters and deeper networks. With these unique feature extraction structures, both two models have good segmentation performance. In addition, the pre-training weights of the two models can accelerate the training of U-Net. So, MobilenetV3 bneck module and VGG16 are chosen to replace the encoder part of the U-Net for investigating and comparing the effects of different feature extraction structure on the model performance. In the experiments, the feature extraction structure of U-Net encoder is replaced by MobilenetV3’s bneck module and VGG16, and the modified models are referred to as MobilenetV3_unet and VGG16_unet respectively. As is shown in the **Table 2**, MPA, MIoU and Dice of VGG16_unet are 1.8%, 1.4% and 0.01 higher than that of original U-Net model; the training time, however, is almost seven times that of the latter. As for as MobilenetV3_unet is concerned, MPA, MIoU and Dice are 1.2%, 1.1% and 0.017 higher than that of U-Net, and the training time is about one half of the latter. Guided by these results, MobilenetV3 bneck module is selected as the feature extraction structure of the proposed model and the following investigation are focused on optimizing the MobilenetV3_unet.

**Table 2:** The evaluation of different feature extraction structures and attention mechanisms.

Evaluation method	Model	MPA (%)	MioU (%)	Dice	Training Time
Evaluation of different feature extraction structures	U-Net	90.95	83.3	0.797	0:37:58
	MobilenetV3_unet	92.15	84.4	0.814	0:18:58
	VGG16_unet	92.75	84.7	0.807	2:15:11
Evaluation of different attention mechanisms	SE_ MobilenetV3_unet	92.15	84.7	0.815	0:22:12
	CBAM_ MobilenetV3_unet	92.75	84.8	0.818	0:22:33
	ECA_ MobilenetV3_unet	92.7	84.7	0.817	0:22:49

**Table 2** also shows the comparison of model performance by adding different attention mechanisms in MobilenetV3_unet. Three different attention mechanism modules such as SE, CBAM and ECA, are added to the same location of MobilenetV3_unet. By comparison, we can see that the addition of attention mechanism improves the segmentation effect, and the MPA and MIoU reaches the highest values after the addition of CBAM. It is 0.6% and 0.4% higher than that of the original MobilenetV3_unet, respectively. Therefore, CBAM attention mechanism module is integrated to construct the optimal model.

Further experiments are performed to investigate the influence of loading MobilenetV3’s pre-training weights on the performance of the model. By comparison, MPA and MIoU with trained weights are 1.85% and 0.4% higher than those without pretrained weights, respectively. Therefore, this paper improves the network by adding pre-training weights.

Through the above comparative experiments, the final model Mobileunet-CC is constructed by replacing the encoder of U-Net network with MobeilNetV3 with pre-training weights, integrating CBAM modules in both encoders and decoders, and adding convolutional layers (Conv) in each double convolutional module of decoder. The ablation experiments are performed and the results of ablation experiments are shown in **Table 3**. In the model, MobileNetV3 is used as the feature extraction network, which utilized techniques such as depthwise separable convolution and global average pooling to provide lower computational cost and efficient feature extraction capability. Additionally, transfer learning is employed to address the problem of small sample learning and accelerate model training. The CBAM added in the encoder can enhance the network’s perception of important features, thereby improving the model’s expressive power and resolution. Integrating CBAM in the U-Net decoder helped the network focus on key features, thereby improving the localization accuracy and detail preservation of segmentation results, ultimately enhancing semantic segmentation in image segmentation tasks. The Conv added in each layer of the U-Net decoder enhances feature fusion, nonlinear modelling, and feature extraction capabilities, thereby improving the segmentation performance and robustness of the decoder. The comparison between the improved model and U-Net shows an increase in pixel accuracy (MPA) by 2.45%, MIoU by 1.7%, and Dice coefficient by 3.2%.

**Table 3:** Results of ablation experiment.

U-Net	MobilenetV3	CBAM	Conv	MPA (%)	MioU (%)	Dice
√	√			92.15	84.4	0.814
√	√	√		92.75	84.8	0.818
√	√	√	√	93.4	85	0.824

#### 3.1.3 Model performance comparison

In order to verify the effectiveness of the proposed Mobileunet-CC on litchi flush segmentation task, a comparison experiment is conducted on litchi flush dataset with five models, namely U-Net, FCN, Deeplabv3, MobileNetV3_unet, and VGG16_unet.

In order to speed up training without compromising accuracy and meet memory limitations, we chose an image size of 512× 512 pixels. The comparison results of different models are shown in **Table 4**. By using encoder and decoding structure, the original U-Net model can extract the features of the input image and retain the details while segmenting the image, with MPA reaching 90.95% and MIoU reaching 83.3%. For FCN models, MPA and MIoU are relatively low since many details are lost after deconvolution and other operations. As for as Deeplabv3 model is concerned, MIoU is relatively low as 75.7%, which may result from the small datasets and training rounds. The MIoU of VGG16_unet reaches a relatively high value of 84.7% since it considers transfer learning, but the pre-trained model has many network parameters and long training time is then required. The MPA and MIoU of the MobilenetV3_unet model are relatively high, but there are still some missing details. For the Mobileunet-CC proposed in this paper, the pixel accuracy reaches 93.4% and the MIoU reaches 85% with the model size reduced to be 28.5MB, indicating that the proposed Mobileunet-CC model has better performance than previous models such as U-Net, Deeplabv3 and VGG16_unet.

**Table 4:** Performance comparison of different segment models.

Model Name	Object	PA (%)	IoU (%)	MPA (%)	MIoU (%)	Model size (MB)
U-Net	background	98.0	97.1	90.95	83.3	33
	flush	83.9	69.5			
FCN	background	98.2	96.2	85.8	77.8	269
	flush	73.4	59.3			
Deeplabv3	background	96.9	95.4	90.65	75.7	320
	flush	78.9	56			
MobilenetV3_unet	background	98.3	97.4	92	84.4	26.6
	flush	87.2	71.4			
VGG16_unet	background	98.5	97.5	92.35	84.7	201
	flush	86.2	71.9			
Mobileunet-CC	background	98.2	97.5	93.4	85	28.5
	flush	88.6	72.5			

**Figure 7:**
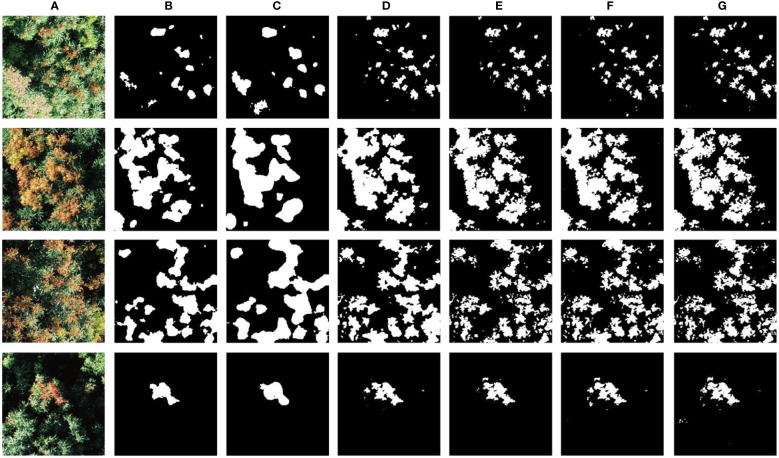
Segmentation results of different models. **(A)**: original images used for segmentation; **(B–G)**: Predicted masks for FCN, Deeplabv3, U-Net, VGG16_unet, MobilenetV3_unet and Mobileunet-CC.

**Figure 7** shows the segmentation results of the litchi flush test image using all models. The first column shows four RGB cropped images (512×512) collected in the litchi Garden of South China Agricultural University in December 2022. Columns 2 through 7 present the predicted masks of FCN, Deeplabv3, U-Net, MobilenetV3_unet, VGG16_unet, and the improved network, respectively. The segmentation effect of FCN and DEEPLABV3 is poor and the leaf contour not obviously segmented. It indicates that the dataset is too small or the model is not suitable for litchi flush segmentation. U-Net, MobilenetV3_unet and VGG16_unet all have a good segmentation performance, but the segmentation of the proposed Mobileunet-CC is more precise, and it is more suitable to segment the small flushes and dense flushes.

### 3.2 Tracking the grown process of winter flush

In order to verify the practical value of the Mobileunet-CC model proposed in this paper, we used the Mobileunet-CC model to track the grown process of winter flush. We used DJI Phantom 4 Pro to take fixed-point aerial images of 15 litchi trees in the Litchi Garden of South China Agricultural University at a height of 10 meters, and the tracking dates were November 20, 2022, December 4, 2022, December 8, 2022, and December 13, 2022, respectively. The collection time was from 10 to 12 a.m., and the weather and temperature on the tracking date were recorded.

**Figure 8:**
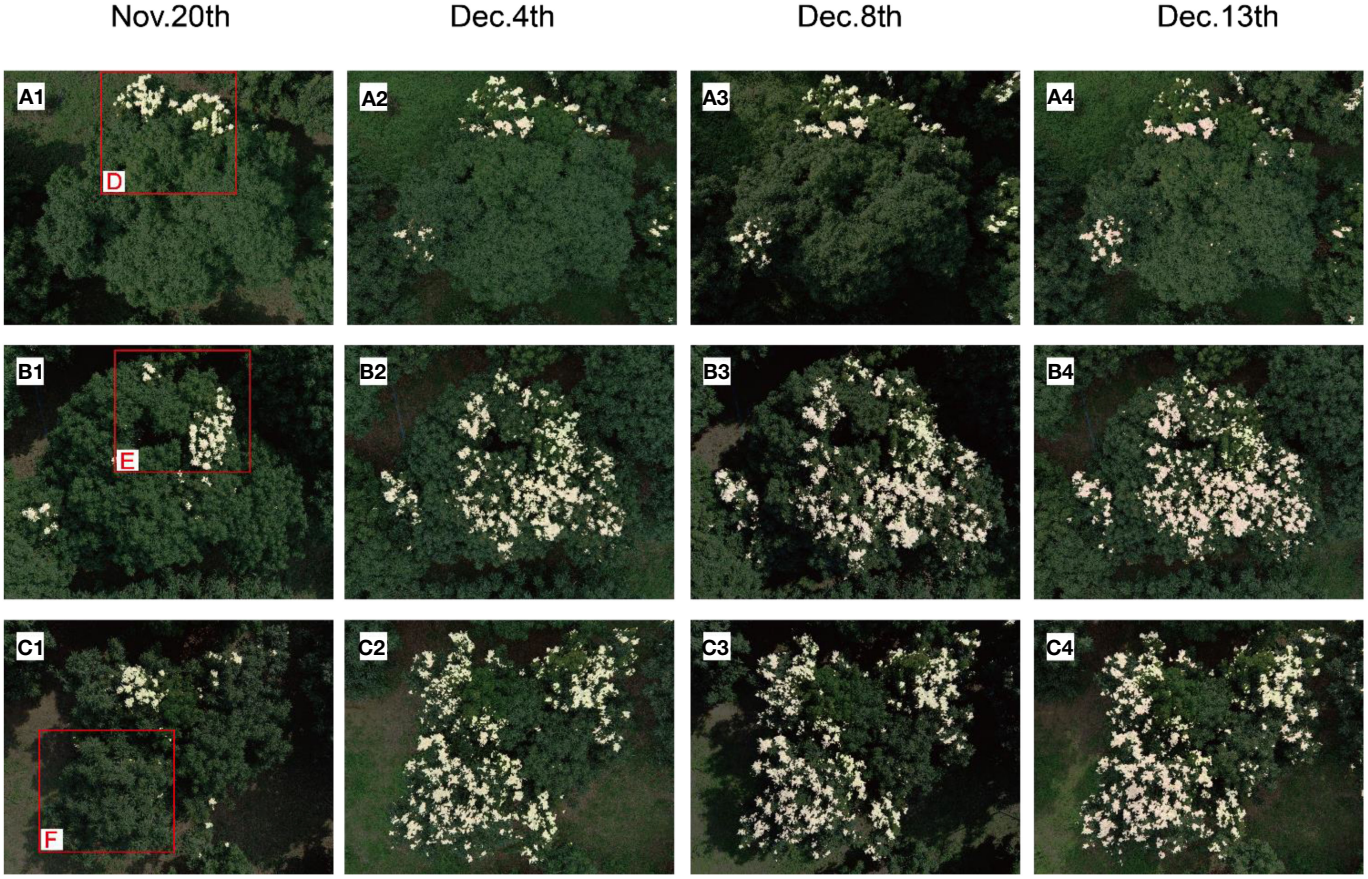
Flush segmentation results of Tree A, B, C. **(A1–A4, B1–B4, C1–C4)** denote the segmentation results on November 20, December 3, December 8, and December 13, respectively.

The Mobileunet-CC model was used to segment the flushes of 15 litchi tree images on four different acquisition dates. The segmentation results of three representative litchi trees A, B and C were superimposed on the original image captured by the UAV to display the evolution process diagram of the flush, as is shown in **Figure 8**. It was observed that the flushes of trees B and C grew in a large range and the colour gradually turned red. As far as tree A, the flushes gradually disappeared in one area but gradually grew in the other two areas. On whole, the overall flush growth for tree A was less than that for trees B and C.

**Figure 9:**
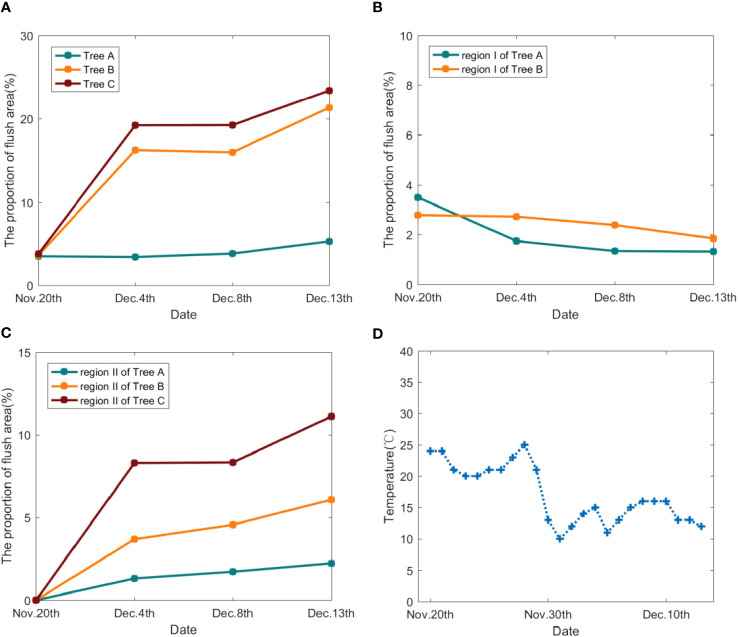
**(A)** POFA of Tree A, B, C; **(B)** POFA of region I for Tree A, B; **(C)** POFA of region II for Tree A, B, C; **(D)** temperature change during the tracking period.

Based on the segmentation effect of the Mobileunet-CC model, we calculated the proportion of flush area (POFA) of three trees at each time for quantitative analysis according to the ratio of the number of pixels in flush to the number of pixels in the canopy, as shown in **Table 5**. **Figure 9A** showed the change curve of POFA in the whole tree. It can be observed that the POFA of litchi trees A, B and C gradually increased, indicating that the autumn and winter shoots have begun to enter the stage of flush development.

**Table 5:** The POFA of total, region I and II for Tree A, B, C.

Tree	Region	POFA (%)			
		November 20th	December 4th	December 8th	December 13th
A	Total	3.53	3.44	5.28	5.30
	I	3.49	1.74	1.34	1.32
	II	0	1.31	1.72	2.23
B	Total	3.65	16.23	15.96	21.33
	I	2.78	2.73	2.38	1.84
	II	0	3.69	4.56	6.08
C	Total	3.82	19.22	19.24	23.39
	I	/	/	/	/
	II	0	8.30	8.33	11.11

The evolution process of flush of each tree was analysed in combination with **Figure 9** and **Table 5**: the POFA of tree B and C increased significantly from November 20 to December 4, from 3.65% and 3.82% to 16.23% and 19.22% respectively. However, the POFA of tree B decreased slightly on December 8, and then continued to increase, while the POFA of tree C was always rising during the tracking period. The POFA of tree A increased slightly, from 3.53% to 5.30%, and had a slight downward trend on December 4. By observing **Figure 8**, it can be found that on December 13, tree B and C had more red and yellowish red leaves and their distribution ranges were wide, with the POFA for 21.33% and 23.39% respectively. If these flushes cannot grow into green leaves in the near future, the tops with flush cannot provide enough nutrients for flower bud differentiation, so it is necessary to continue tracking and carry out effective tip control operations. The flush area of tree A was more concentrated and less distributed, so we only need to track the flush area. By continuously monitoring the flushes of each litchi tree, the changes of the location, range and area of the flushes can be accurately obtained, which provides information and guidance for the subsequent control operation.

**Figure 10:**
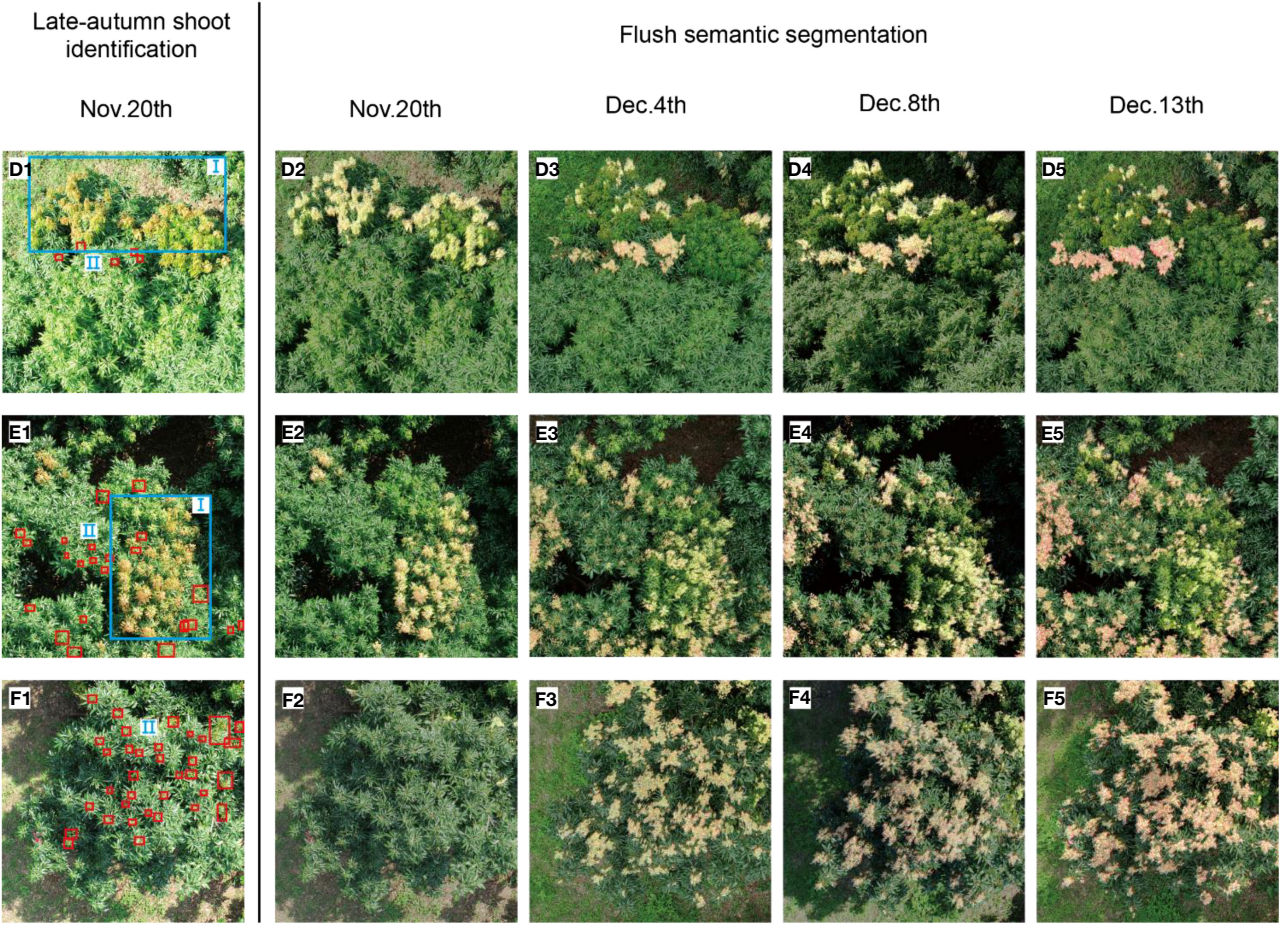
**(D1, E1, F1)** show late-autumn shoots in regions D, E and F of Tree A, B and C on November 20, 2022, respectively (red boxes represents late-autumn shoots identified by AI model). **(D2–D5, E2–E5, F2–F5)** denote the visual image of the evolution process of flushes in region D, E and F on November 20, December 3, December 8, and December 13, respectively.

In order to further show the evolution of flush and analyse the reasons for the change of flush, we carried out accurate analysis of regional branches. In **Figure 8** A1, B1, C1, three representative regions shown in the red box were selected for research and named as region D, E and F respectively. The region D, E and F were enlarged and displayed in **Figure 10**. Firstly, YOLOv5-SBiC late-autumn shoot identification model (Liang et al., 2023) proposed by our laboratory was used to identify the images of the late-autumn shoots from the images of these three regions on November 20. The recognition results are shown in the red boxes in D1, E1 and F1 in **Figure 10**. Then, through observation, the regions D, E and F were further subdivided into region I (flushes gradually growing into mature leaves) and region II (late-autumn shoots growing into flushes), which were the blue box part and the small red box part in D1, E1 and F1 of **Figure 10** respectively. Finally, the POFA in the two regions was calculated, as shown in **Table 5**, and its change curve was shown in **Figures 9B**, **9C**.

The evolution process of flushes in region I and II of trees A, B and C are analysed. In tree A, POFA of region I decreased from 3.49% to 1.74% in the period of November 20 to December 4, and then slowly decreased to 1.32%. The POFA in region II increased from 0% to 2.23%, and the growth of flushes was small. In the region where flushes grew at December 3, December 8, and December 13, a few late-autumn shoots can be identified by AI model on November 20 (see **Figure 10D1**). In other word, the flushes in region II grow from the late-autumn shoots, which can be recognized by YOLOv5-SBiC model (Liang et al., 2023). Shoots control operation should be carried out before they grow into flushes.

For tree B, POFA of I region decreased from 2.78% to 1.84% between the period of November 20 to December 13. The flushes on December 13 were in the yellowish green stage, which can be quickly changed to green without intervention. As far as region II is concerned, the POFA increased from 0% to 6.08% at this time period and a sudden increase in flush was observed on December. This happened because the late-autumn shoots spread out into flushes when encountering low-temperature environments during winter. It is supported by the facts that a large number of late-autumn shoots were identified in region II by the AI model (see **Figure 10E1**) and there was a sudden drop in temperature from November 28 to November 31 (see **Figure 9D**).

No flushes were observed in region II of Tree C on November 20, as is shown in **Figure 10F1**. On December 3, however, the flush increased significantly and reached a high POFA of 8.30% due to the temperature drop during November 28 to November 31. The relatively low temperature environments rendered the late-autumn shoots quickly grow into flushes, cover the entire canopy and the POFA further increased to 11.11% on December 13. Since the late-autumn shoots can be identified by AI model obtained on November 20 (see **Figure 10F1**), one can accurately predict the generation of flush in the future time according to the temperature prediction by the meteorological department.

Through the above experiments and analysis, it can be concluded that the Mobileunet-CC model proposed in this paper had good segmentation effect, and can be practically applied to the flush growth monitoring of the whole litchi tree and regional branches. Secondly, the Mobileunet-CC model combined with YOLOv5-SBiC late-autumn shoots identification model proposed in our research group (Liang et al., 2023), can be used to predict the generation of flush in the early stage and then analyse the evolution process of flushes in a particular region of tree according to temperature changes and accurate statistical data. In other words, UAV remote sensing combined with the AI models provides guiding significance for the management of flush in the early, middle and late stages of litchi orchards.

## 4 Conclusions

An efficient approach based on UAV remote sensing and deep learning technology is developed to segment the flush of litchi tree and then used to estimate the area and monitor the growth process of the winter flush. We conducted experiments to evaluate six deep neural networks for semantic segmentation of litchi flush images based on UAV remote sensing images and the results demonstrates that semantic segmentation is very suitable for separating and extracting litchi flushes from UAV remote sensing images. First, we preprocessed the RGB images collected by the UAV and build the flush dataset for constructing segmentation model. Then, we propose a lightweight semantic segmentation model of Mobileunet-CC, which is based on U-Net network with the encoder replaced by MobeilNetV3 backbone network and CBAM modules integrated in both encoders and decoders. The experimental results showed that the improved semantic segmentation model can segment litchi flush accurately and efficiently, with MPA increased from 90.95% to 93.4%, MIoU increased from 83.3% to 85% and model size reduced by 15% to 28.5MB.

In order to verify the practical value of the segmentation model proposed in this paper, the segmentation model was also used to segment the flushes of winter litchi tree growth images tracked by UAV. Two growth processes of litchi flushes (late-autumn shoots growing into flushes and flushes growing into mature leaves) were quantitatively analysed according to different branch regions of the same litchi tree by combining the identification model of late-autumn shoots and the change of flush area. The method can quickly and accurately segment the flushes of litchi trees. It is used to monitor the growth change of flushes and predict the generation of flush in the next stage by combining the extracted flush information with the quantitative analysis and trend of temperature change. The results demonstrate that UAV remote sensing combined with the AI models can be used to predict the generation of flush from late-autumn shoots according to the temperature prediction by the meteorological department. The results presented in this paper provide the possibility of accurate analysis for branch management and flush control operation in litchi orchard, and help to reduce the labour cost of litchi orchard management.

## Data availability statement

The raw data supporting the conclusions of this article will be made available by the authors, without undue reservation.

## Author contributions

SB: Conceptualization, Formal analysis, Methodology, Project administration, Software, Writing – original draft, Writing – review & editing. JL: Conceptualization, Data curation, Investigation, Supervision, Writing – review & editing. TL: Conceptualization, Formal analysis, Investigation, Visualization, Writing – review & editing. CL: Resources, Validation, Writing – review & editing. JXZ: Data curation, Writing – review & editing. WG: Validation, Writing – review & editing. BH: Resources, Writing – review & editing. YuL: Project administration, Writing – review & editing. YoL: Conceptualization, Funding acquisition, Project administration, Supervision, Writing – review & editing. JZ: Conceptualization, Funding acquisition, Project administration, Supervision, Writing – review & editing.
